# Therapeutic Administration of Broadly Neutralizing FI6 Antibody Reveals Lack of Interaction Between Human IgG1 and Pig Fc Receptors

**DOI:** 10.3389/fimmu.2018.00865

**Published:** 2018-04-24

**Authors:** Sophie B. Morgan, Barbara Holzer, Johanneke D. Hemmink, Francisco J. Salguero, John C. Schwartz, Gloria Agatic, Elisabetta Cameroni, Barbara Guarino, Emily Porter, Pramila Rijal, Alain Townsend, Bryan Charleston, Davide Corti, Elma Tchilian

**Affiliations:** ^1^The Pirbright Institute, Woking, United Kingdom; ^2^School of Veterinary Medicine, University of Surrey, Guilford, United Kingdom; ^3^Humabs BioMed SA, Bellinzona, Switzerland; ^4^School of Veterinary Sciences, University of Bristol, Langford, United Kingdom; ^5^Weatherall Institute for Molecular Medicine, University of Oxford, Oxford, United Kingdom

**Keywords:** influenza, anti-stem antibody, pig, Fc receptor, FI6, enhanced disease

## Abstract

Influenza virus infection is a significant global health threat. Because of the lack of cross-protective universal vaccines, short time window during which antivirals are effective and drug resistance, new therapeutic anti-influenza strategies are required. Broadly, cross-protective antibodies that target conserved sites in the hemagglutinin (HA) stem region have been proposed as therapeutic agents. FI6 is the first proven such monoclonal antibody to bind to H1–H16 and is protective in mice and ferrets. Multiple studies have shown that Fc-dependent mechanisms are essential for FI6 *in vivo* efficacy. Here, we show that therapeutic administration of FI6 either intravenously or by aerosol to pigs did not reduce viral load in nasal swabs or broncho-alveolar lavage, but aerosol delivery of FI6 reduced gross pathology significantly. We demonstrate that pig Fc receptors do not bind human IgG1 and that FI6 did not mediate antibody-dependent cytotoxicity (ADCC) with pig PBMC, confirming that ADCC is an important mechanism of protection by anti-stem antibodies *in vivo*. Enhanced respiratory disease, which has been associated with pigs with cross-reactive non-neutralizing anti-HA antibodies, did not occur after FI6 administration. Our results also show that *in vitro* neutralizing antibody responses are not a robust correlate of protection for the control of influenza infection and pathology in a natural host model.

## Introduction

Influenza virus infection and immunization induce protective antibody responses. A major part of the antibody response is directed at the hemagglutinin (HA) glycoprotein. Influenza HA is composed of two domains: the immunodominant globular head, which is strain-specific and the stalk which is relatively conserved within each subtype. Seasonal immunization induces antibodies predominantly against the globular head which neutralizes the immunizing strain very effectively, but escape variants rapidly emerge and are responsible for antigenic drift. In the past decades, influenza-neutralizing antibodies that target conserved sites in the HA stem of influenza A viruses (IAVs) have been described and these show cross-reactivity between group 1 and 2 viruses (1–7). Anti-stem antibodies are less potent at direct viral neutralization as compared to anti-head antibodies, but they mediate protection *in vivo* through Fc-dependent effector functions, which can be assessed *in vitro* by measuring antibody-dependent cellular cytotoxicity (ADCC), complement-dependent cytotoxiticy, or antibody-dependent cellular phagocytosis (ADCP) (4, 8, 9). FI6 was the first proven broadly neutralizing antibody to be described, capable of recognizing the HAs of all 16 subtypes and neutralizing both group 1 and 2 IAVs (4). Passive transfer of FI6 conferred protection in mice and ferrets. It has been proposed that such broadly cross-reactive antibodies might have potential as therapeutic agents for treatment of severe influenza and several are tested in clinical trials (10, 11).

A potential problem of developing such antibodies as immune therapeutics is enhanced respiratory disease and increased pathology, associated with immune complexes of low avidity or non-neutralizing antibodies. Vaccine-associated enhanced respiratory disease (VAERD) has been observed in pigs when heterologous IAV infection occurs after immunization with mismatched whole inactivated vaccine (12–15). VAERD was associated with the presence of high titer cross-reacting non-neutralizing antibodies targeting the conserved stem domain at a site adjacent to the fusion peptide. In the absence of neutralizing antibodies against the globular head of H1N1pdm09, stem antibodies were associated with increased virus infection of Madin–Darby canine kidney (MDCK) cells *in vitro* and enhanced membrane fusion (16).

As both pigs and humans are readily infected with IAVs of similar subtype, the pig is an appropriate model for investigating both swine and human disease. Like humans, pigs are outbred, and physiologically, anatomically, and immunologically similar to humans. The porcine lung also resembles the human in terms of its physiology, morphology, and distribution of receptors bound by IAV (17, 18). Here, we used the pig influenza model to test whether therapeutic administration of FI6 would reduce or enhance disease.

## Materials and Methods

### Animals and Influenza Virus Challenge

**Figure 1:**
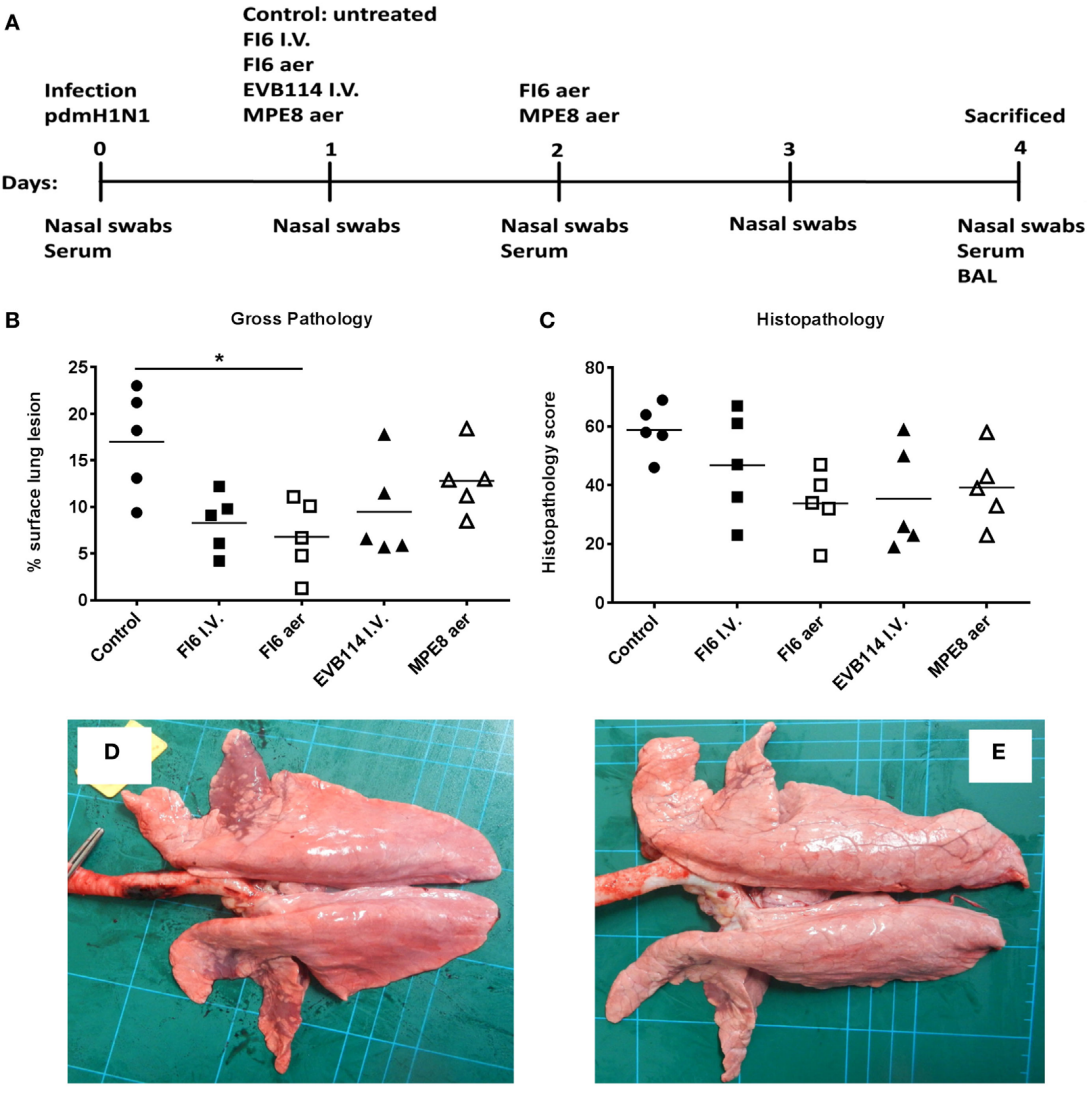
Experimental design and lung pathology. Pigs were infected with A/sw/Eng/1353/09 and received the indicated antibodies either by the intravenous route at 1 day post infection (dpi) or by aerosol at 1 and 2 dpi **(A)**. The animals were sacrificed at 4 dpi and lungs scored for appearance of gross **(B)** and histopathologcial lesions **(C)**. Each data point represents an individual within the indicated group and lines represent the mean. * denotes significant difference from the control group (*P* < 0.05). Representative gross pathology of a pig from control group **(D)** and FI6 aerosol **(E)**.

Animal experiments were approved by the Pirbright Institute ethics committee, according to the UK Animal (Scientific Procedures) Act 1986. Five- to six-week-old landrace cross, female pigs were obtained from a commercial high health status herd. Pigs were screened for absence of IAV infection by matrix (M) gene real-time quantitative reverse transcriptase polymerase chain reaction (qRT-PCR) (19) and antibody-free status was confirmed using HA inhibition with 4 swine IAV antigens—pandemic H1N1, H1N2, H3N2, and avian-like H1N1. Pigs weighed between 9 and 12 kg. All pigs were challenged with 1 × 10^7^ plaque forming units (PFU) of A/sw/Eng/1353/09 (pdmH1N1) influenza virus strain. The pigs were inoculated by the intra-nasal route using a mucosal atomization device, MAD300 (Wolfe Tory Medical) with 2 ml of virus administered to each nostril. The virus was propagated in MDCK cells. The challenged pigs were randomly divided into five groups of five animals and received the following antibodies (experimental design in Figure 1A). (1) Control group—no treatment; (2) 15 mg/kg of FI6 antibody intravenously (FI6 I.V.) in the ear vein at 1 day post infection (dpi); (3) 1.5 mg/ml FI6 antibody administered by aerosol (FI6 aer) using InnosSpire Mini (Philips Respironics http://evergreen-nebulizers.co.uk/respironics/innospire_mini.html) with Aerogen mesh reservoir with an airspeed of 2 L/min at 1 and 2 dpi; (4) 15 mg/kg of EVB114 antibody I.V. in the ear vein at 1 dpi; and (5) 1.5 mg/kg of the MPE8 antibody by aerosol at 1 and 2 dpi as described above. All antibodies were provided by Humabs BioMed. They were produced in Chinese hamster ovary cells, affinity-purified using HiTrap Protein A columns (GE Healthcare) followed by desalting using HiTrap Fast desalting columns (GE Healthcare). The final product were sterilized by filtration through 0.22 µm filters and stored at +4°C until use. Antibodies were diluted in phosphate buffered saline (PBS) to the desired concentration before administration. Animals were monitored by observing demeanor, appetite, and respiratory signs, such as coughing and sneezing.

### Gross Pathology and Histopathological Scoring of Lung Lesions

Animals were humanely killed 4 dpi with an overdose of pentobarbital sodium anesthetic. At *post mortem*, the lungs were removed and digital photographs taken on the dorsal and ventral aspects. Macroscopic pathology scoring was performed blind using Nikon-NIS Br software to determine the proportion of the total surface area of each lung lobe affected by typical influenza-like gross lesions. Five lung tissue samples per animal from the right lung (2 pieces from apical lobe, 1 from the medial, 1 from the diaphragmatic, and 1 from the accessory) were collected into 10% neutral buffered formalin for routine histological processing at the University of Surrey. Formalin fixed tissues were paraffin wax-embedded and 4-µm sections were cut and routinely stained with hematoxylin and eosin. Histopathological changes in the stained lung tissue sections were scored by a veterinary pathologist blinded to the treatment group. Lung histopathology was scored using five parameters (necrosis of the bronchiolar epithelium, airway inflammation, perivascular/bronchiolar cuffing, alveolar exudates, and septal inflammation) scored on a five-point scale of 0–4 and then summed to give a total slide score ranging from 0 to 20 and a total animal score from 0 to 100. Scoring criteria were based upon a previously published method (20).

### Tissue Sample Processing

Four nasal swabs (NS) (two per nostril) were taken at 0, 1, 2, 3, and 4 dpi. The swabs were placed into 2 ml of virus transport medium comprising tissue culture medium 199 (Sigma-Aldrich) supplemented with 25 mM 4-(2-hydroxyethyl)-1-piperazineethanesulfonic acid (HEPES), 0.035% sodium bicarbonate, 0.5% bovine serum albumin, penicillin 100 IU/ml, streptomycin 100 µg/ml, and nystatin 0.25 µg/ml, vortexed, centrifuged to remove debris, and stored at −80^o^C for subsequent virus titration. Serum samples were collected at the start of the study (prior to challenge) and at 2 and 4 dpi. For Fc binding and ADCC assays, blood from healthy humans and uninfected pigs was used. Heparinized blood samples were diluted 1:1 in PBS before density gradient centrifugation. PBMC were harvested from the interface, washed and red blood cells lysed with ammonium chloride lysis buffer, washed again, and used in Fc binding and ADCC assays described below. Broncho-alveolar lavage (BAL) was collected from the entire left lung with 150 ml of virus transport medium (described above). BAL samples were centrifuged at 300 × *g* for 15 min, supernatant was removed, aliquoted, and frozen for antibody analysis.

### Virus Titration

Viral titers in nasal swabs and BAL were determined by plaque assay on MDCK cells. Duplicate samples were 10-fold serially diluted in Dulbecco’s modified Eagle’s medium and 100 µl of each dilution added to confluent MDCK cells in 12-well tissue culture plates. After 1 h, the plates were washed and overlayed with 2 ml 1:32% (w/v) agarose:medium. Plates were incubated at 37°C for 48 h, plaques visualized by staining the monolayer with 0.1% (v/v) crystal violet, and enumerated. RNA was extracted using the QIAamp viral RNA mini kit (Qiagen) according to the manufacturer’s protocol and viral titers in nasal swabs and BAL fluid was also determined by qRT-PCR amplification of the M gene using PCR conditions as previously described (21). Forward primer sequence AGA TGA GTC TTC TAA CCG AGG TCG, reverse primer sequence TGC AAA GAC ACT TTC CAG TCT CTG, and probe sequence FAM-TCA GGC CCC CTC AAA GCC GA-TAMRA.

### Enzyme-Linked Immunosorbent Assay (ELISA)

Human IgG1 antibody levels in serum and BAL fluid were determined by IgG1 Ready-SET-Go! ELISA (Affymetrix, eBioscience) according to the manufacturer’s instructions. After heat inactivation (56^°^C for 30 min) samples were diluted 1:40 (serum) and 1:2 (BAL fluid). Influenza-specific human Ab titers in serum and BAL fluid were determined by ELISA as previously described (20) with the following modifications. The IgG ELISA was performed in 96-well ELISA plates (BD Biosciences) coated with 1 × 10^6^ PFU/ml of A/swine/England/1353/09 over night at 4°C. Twofold dilutions of BAL fluid samples or serum (heat inactivated for 30 min at 56°C) were added, starting from 1:2 or 1:10 dilution, respectively. Binding of influenza-specific Abs was detected using a monoclonal anti-human IgG (hIgG) (Fc) (Biorad) and 3,3′,5,5′-tetramethylbenzidine (TMB) substrate (BioLegend). Optical density (OD) readings were taken at 450 and 570 nm (wavelength correction). Ab values were expressed as endpoint titers defined as the highest dilution at which the OD was higher than twice the background OD.

### Fc Binding

To determine if FI6 was able to bind pig Fc receptors FI6, MPE8, and serum from influenza negative and immune (14 dpi) animals were incubated at 37°C for 1 h with and without influenza virus. Human and pig PBMC were added and incubated for a further hour at 4°C. Human PBMC were stained with near-infrared fixable Live/Dead (Invitrogen) and anti-hIgG AF488 (HP6017, Biolegend) for 20 min at 4°C. Pig PBMC were stained with near-infrared fixable Live/Dead (Invitrogen), CD3 AF647 (BB23-8E6-8C8, BD), CD8α Pe (76-2-11, BD) anti-hIgG, or anti-pig IgG FITC (BIO-RAD). Samples were run on a BD LSR Fortessa and data analyzed using FlowJo (Treestar).

### Entry Microneutralization Assay

Serum and BAL fluid were heat inactivated at 56°C for 30 min, serially diluted 1:2 in 50 µl PBS, starting dilution 1:40 for serum and 1:4 for BALF, before addition of 50 µl green-fluorescent protein (GFP)—H1 virus diluted in virus growth medium (22). Following incubation for 2 h at 37°C 3 × 10^4^ indicator MDCK-sialyltransferase (SIAT1) cells were added in a volume of 100 µl virus growth medium without trypsin and incubated overnight at 37°C. Plates were fixed using 4% paraformaldehyde and GFP fluorescence intensity (FI) was measured at an excitation of 483 nm and an emission of 515 nm. Serum and BALF from animals 14 days post influenza challenge and purified FI6 antibody were included as positive controls.

### ADCC Assay

Madin–Darby canine kidney–2,6-sialyltransferase (SIAT1) stably transduced with the lentiviral vector pHR-SIN engineered to express the full-length open-reading frame of HA from A/Eng/195/2009 were used as target cells for the ADCC assay (22). The HA from A/Eng/195/2009 differs by a single exposed residue at D222G from the Eng/1353 that was used to challenge the pigs. MDCK-HA cells were seeded in round bottom 96-well plates and incubated with different dilutions of heat-inactivated serum (1:10, 1:20, 1:40, 1:80, or 1:160) or with different amounts of antibody (FI6 or MPE8) for 10 min at 37 C. After that freshly isolated human or pig PBMCs from healthy donors or animals respectively, were added in a 20 to 1 E:T ratio to the 96-well plates and incubated for 4 h at 37°C. MDCK-HA and PBMC were cultured in serum-free AIM-V medium (Life Technologies, UK). At the end of the incubation period, 100 µl of cell-free supernatant was transferred into a flat bottom 96-well plate and the lactate dehydrogenase (LDH) release is measured using the Cytotoxicity Detection Kit from Roche according to the manufacturer’s instructions. The absorbance was measured at 490 and 620 nm in a plate reader. When the purified antibodies, MPE8 and FI6, were used as the percentage of cytotoxicity on the *Y*-axis was calculated with the formula: [Sample at each immune antibody dilution with target cells and PBMC minus control antibody at the same dilution with target cells and PBMC] divided by [maximum release of target cells and PBMC in the presence of detergent minus control target and effector spontaneous release without antibody] × 100. In assessing the ADCC activity of serum samples, the percentage of cytotoxicity was calculated as described above, but using the naïve sera (corresponding dilution to sera of immunized pigs to calculate the spontaneous release).

### Statistical Analysis

One-way non-parametric ANOVA (Kruskal–Wallis) with Dunn’s post-test for multiple comparisons was performed using GraphPad Prism 6.

## Results

### Lung Pathology and Viral Load After Antibody Administration

**Figure 2:**
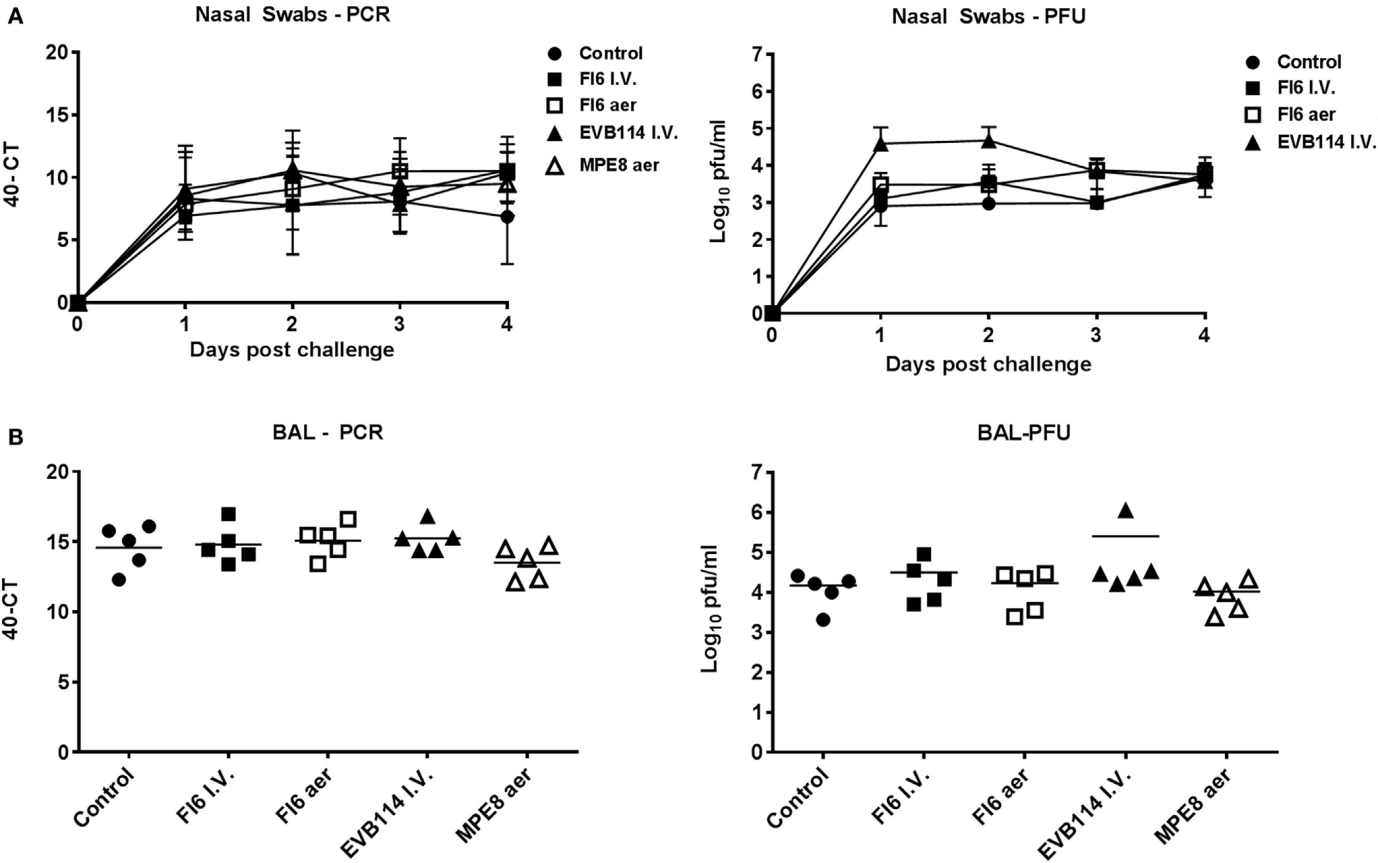
Viral load in nasal washes and broncho-alveolar lavage (BAL). Pigs were infected with A/sw/Eng/1353/09 and administered the indicated antibodies either by the I.V. route (I.V. solid symbols) or by aerosol (aer, hollow symbols). Nasal swabs (NS) were taken at 0, 1, 2, and 3 day post infection (dpi) and pigs sacrificed at 4 dpi. Viral titers in the nasal swabs **(A)** and BAL **(B)** were determined by plaque forming assay or real-time quantitative reverse transcriptase polymerase chain reaction. Each data point represents an individual within the indicated group and bars represent the mean.

In order to determine the therapeutic effect of FI6 antibody in the pig influenza model, FI6 was administered I.V. at 15 mg/kg 1 dpi. The ebola virus-specific antibody, EVB114 was used as a control and delivered at the same concentration I.V. (23). We also administered FI6 as an aerosol (aer) as this route of delivery is highly efficient in targeting the respiratory tract, which is the site of entry and infection of IAV (20, 24–28). We administered 10 times less FI6 by aerosol (1.5 mg/kg) at 1 and 2 dpi. As a control for the aerosol delivery we used MPE8, which is a broadly neutralizing antibody for human respiratory syncytial virus, human metapneumovirus, bovine RSV, and pneumonia virus of mice but not IAV (29) (Figure 1A). All of the mAbs were monoclonal, fully human IgG1. The clinical signs observed were mild and none of the pigs developed moderate or severe disease. The control group showed the most severe gross and histopathology (Figure 1B–E). A reduction in the gross and histopathology score was observed in all the mAb-treated groups. However, this reduction was statistically significant only in gross pathology for the FI6 aer group. Interestingly despite the reduced lung pathology, there were no differences in viral load in nasal swabs at 1, 2, 3, and 4 dpi (Figure 2A) or in the BAL at the time of sacrifice at 4 dpi (Figure 2B) as determined by plaque forming assays and PCR. This is in contrast with previous studies in mice and ferrets, where FI6 administration significantly reduced viral load in the lungs (4).

These results indicate that administration of therapeutic FI6 to pigs did not reduce viral load in NS or BAL, but also it did not exacerbate disease as previously shown with anti-stem antibody (16). The mAb-treated groups showed reduced pathology, although the reduction was significant only for the aerosol FI6 group. The reduced pathology is also observed using control antibodies, a finding that might be related to the anti-inflammatory and immunomodulatory activities of human IgG1 Fc. Indeed, it has been shown that the anti-inflammatory activity of human intravenous immunoglobulin is dependent on sialylation of the N-linked glycan of the IgG1 Fc fragment (30, 31).

### Influenza Binding and Neutralizing Activity of Administered Antibodies

**Figure 3:**
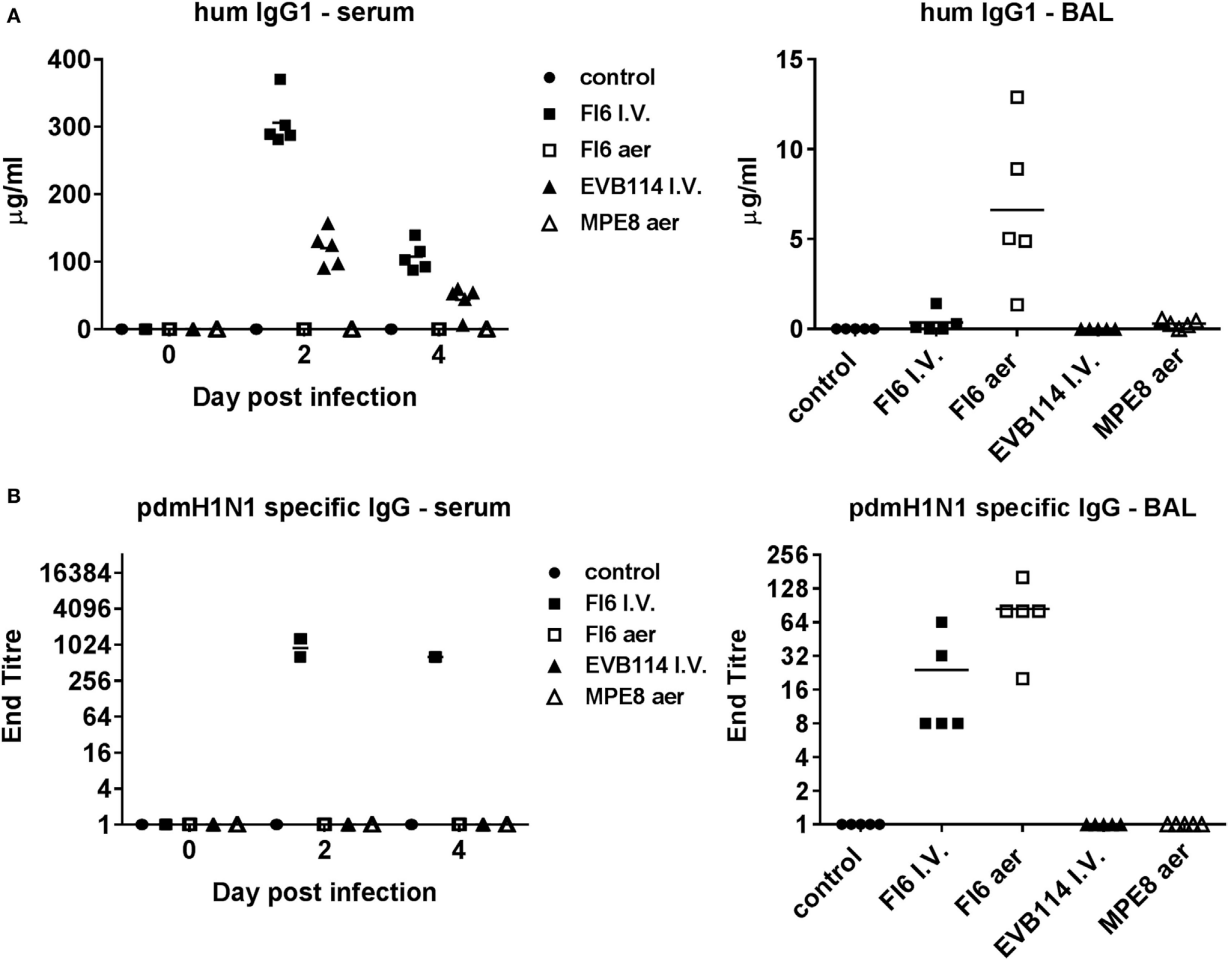
Mucosal and systemic IgG responses following administration of antibodies. Human IgG1 **(A)** and pdmH1N1-specific IgG **(B)** titers in serum at 0, 2, and 4 day post infection (dpi) and in broncho-alveolar lavage at 4 dpi. Note for pdmH1N1-specific antibody in serum—all five pigs had titers of 1:640 at 4 dpi, while two animals at 1:1,024, and three at 1:640 at 2 dpi.

Enzyme-linked immunosorbent assay for human IgG1 confirmed that all antibodies were delivered successfully, albeit the control EVB114 was detected at a lower concentration in the serum perhaps due to differences in the catabolic rates of this mAb. The mAb concentrations declined at 4 dpi compared to 2 dpi, but were still ~107 μg/ml for FI6 and ~45 μg/ml for EVB114 (Figure 3A). Aerosol administration of FI6 and MPE8 did not result in detectable quantities of mAbs in the serum. However, mAbs were detected in BAL, with ~6.5 µg/ml for FI6 and 0.5 µg/ml for MPE8 measured at 4 dpi, 2 days after the last aerosol administration, most likely indicating that the mAbs are catabolized rapidly after aerosol delivery (Figure 3A). Furthermore I.V. FI6 delivery resulted in the presence of ~0.33 µg/ml in the BAL 4 days after the administration of the antibody, approximately 20-fold less as compared to the aerosol FI6 group. To further confirm the presence and specificity of FI6 after delivery, virus-specific ELISA was performed with the challenge virus. As expected influenza-specific human mAb was detected in serum after FI6 I.V. delivery at both 2 and 4 dpi, while in BAL a higher titer was seen after aerosol (1:84) compared to FI6 I.V. administration (1:24) (Figure 3B).

**Figure 4:**
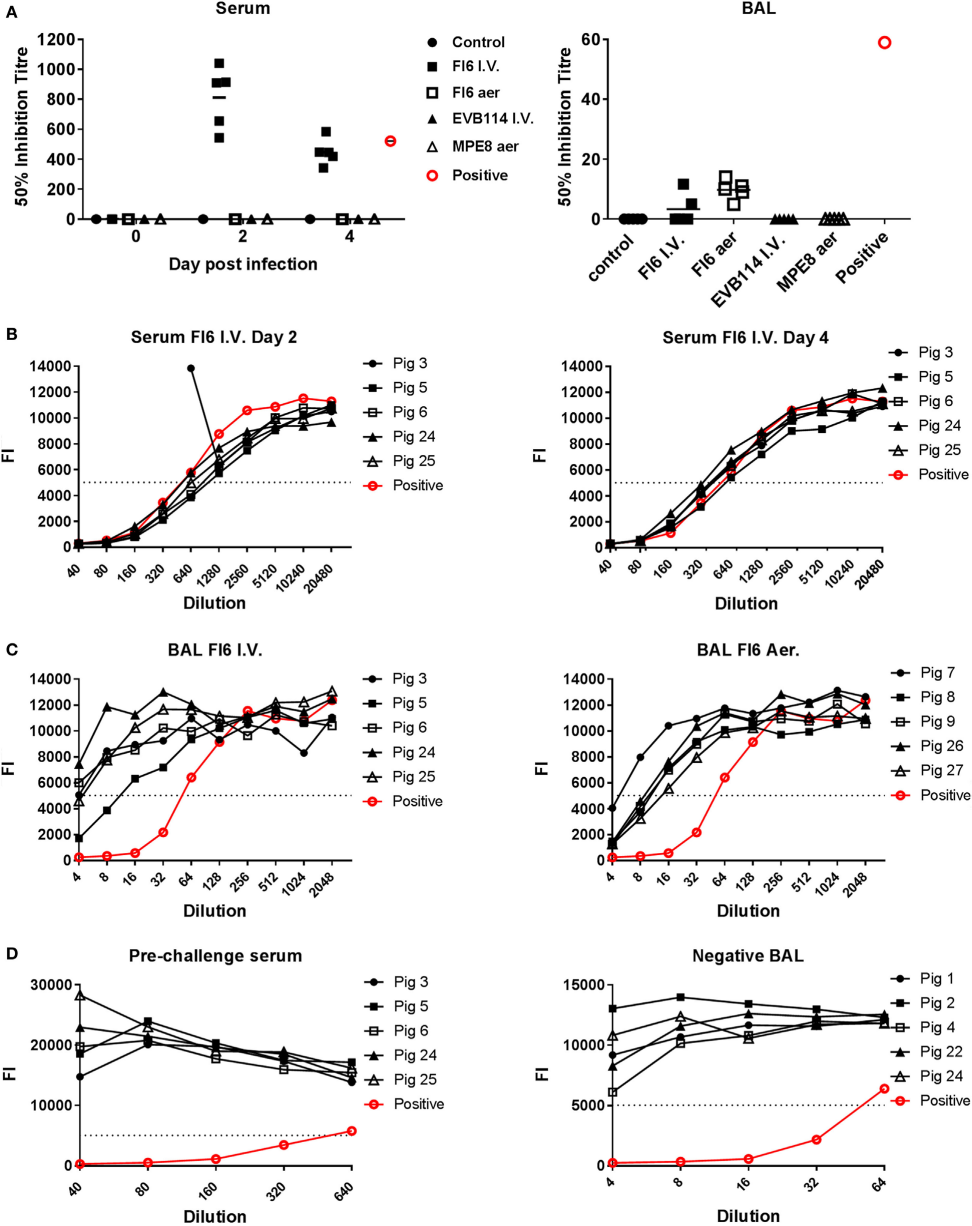
Entry neutralization activity of serum and broncho-alveolar lavage (BAL) following administration of antibodies. **(A)** Individual 50% inhibition titers in the serum at 0, 2, and 4 day post infection (dpi) and BAL at 4 dpi. **(B)** Neutralizing antibody response measured in the serum of the pigs following FI6 I.V. administration at 2 and 4 dpi. **(C)** Antibody response in the BAL of animals given FI6 I.V. or by aerosol at 4 dpi. **(D)** Negative sera from pre-challenge samples and negative BAL from control animals are shown alongside positive control. The dashed line represents the 50% inhibition titer and FI the fluorescence intensity of green-fluorescent protein. The neutralizing titer of serum and BAL from animals infected with the same A/sw/Eng/1353/09 virus and sacrificed at 14 dpi is shown in red.

To determine whether the FI6 in the serum and BAL were still able to neutralize the virus, which might explain the lack of reduction of viral titer, we performed entry virus neutralization (22). The serum from the FI6 I.V. group was neutralizing at both 2 and 4 dpi with a mean 50% inhibitory titer of 1:812 at 2 dpi and 1:448 at 4 dpi (Figures 4A,B), comparable to control immune pig serum. In the BAL of the FI6 aerosol group the mean 50% inhibitory titer was 1:10 at 4 dpi and in the FI6 I.V. group 1:3.4 (although only 2 out of the 5 animals had positive results) (Figures 4A,C). The neutralization values for the BAL were lower than a control BAL fluid (1:640) from a pig sacrificed 14 days post challenge with the same virus. No neutralization was detected in the animals receiving control antibodies or in the untreated controls. Pre-challenge sera from FI6-treated animals and BAL from control animals did not show any neutralizing activity (Figure 4D).

Overall these results indicate that the mAbs were successfully delivered and retained their influenza binding and neutralizing activity as measured *in vitro*.

### Fc Binding and ADCC

**Figure 5:**
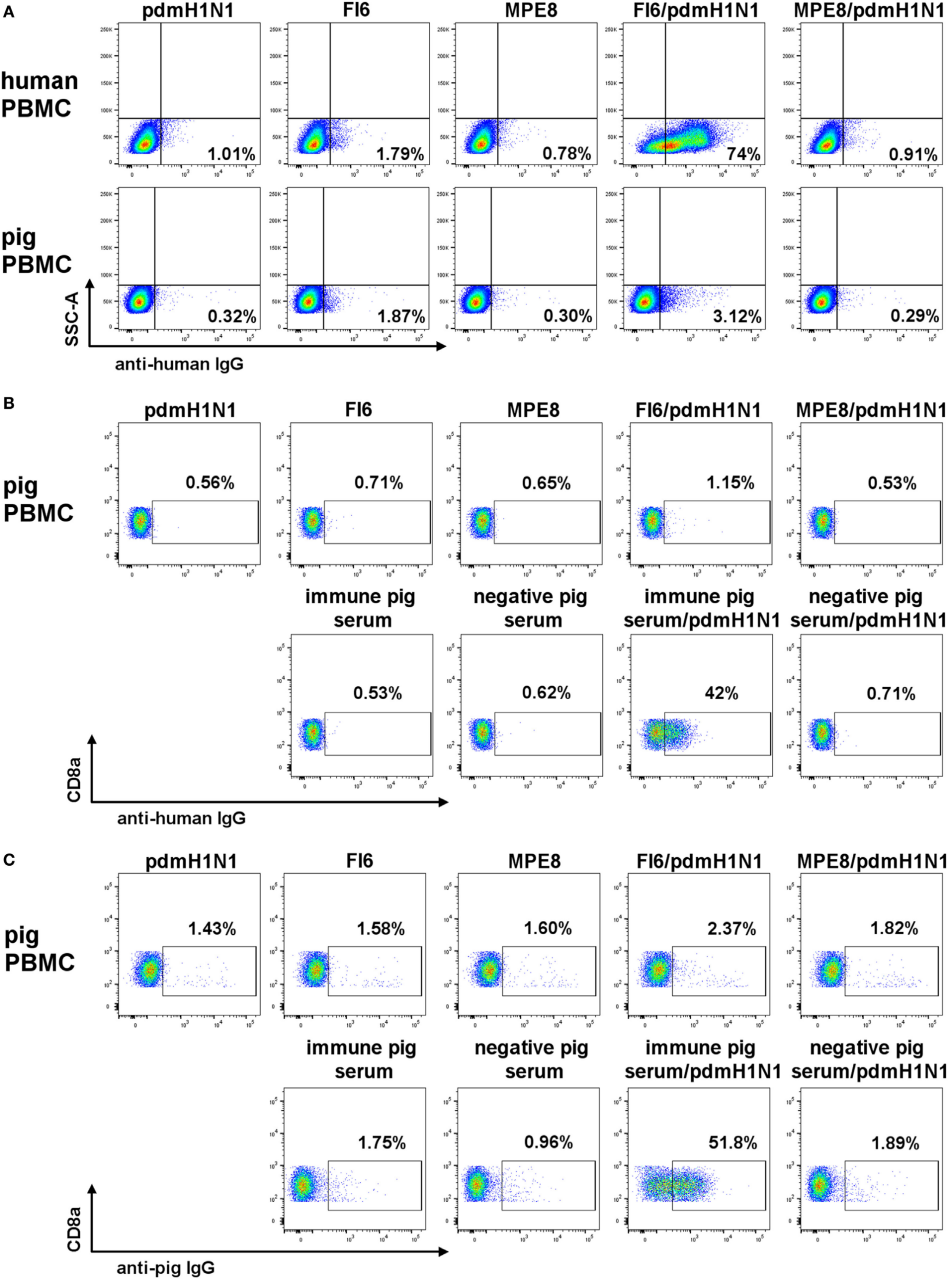
FI6 binding to human and pig Fc receptors. Antibody and pdmH1N1 virus were pre-incubated for 1 h at 37°C and then added to either human or pig PBMC. **(A)** Gated on live cells, singlets, and SSCA versus IgG FITC. **(B,C)** Gated on live cells, singlets, and CD3^−^CD8α^+^ IgG FITC.

As it has been shown convincingly that most broadly neutralizing anti-IAV mAbs mediate their *in vivo* effect through antibody effector functions (4, 9, 32), we next asked whether the FI6 or human IgG1 can bind pig FcR and mediate ADCC. Fc binding was assessed after pre-incubating the mAbs with pdmH1N1 virus in order to form immune complexes. As expected human lymphocytes bound FI6 with 74% of the lymphocytes stained compared to less than 2% for the controls (Figure 5A). In contrast, minimal binding of FI6/pdmH1N1 to pig PBMC was detected. A more detailed analysis was performed by gating on pig NK cells, defined as CD3^−^CD8^+^ (Figures 5B,C), which bound immune pig serum pre-incubated with pdmH1N1, but bound very little FI6 (51.8% for immune pig serum versus 2.37% for FI6). Similar results were obtained after detection of immune complexes with secondary anti-hIgG, indicating that this antibody could bind the pig Ig (Figure 5C).

**Figure 6:**
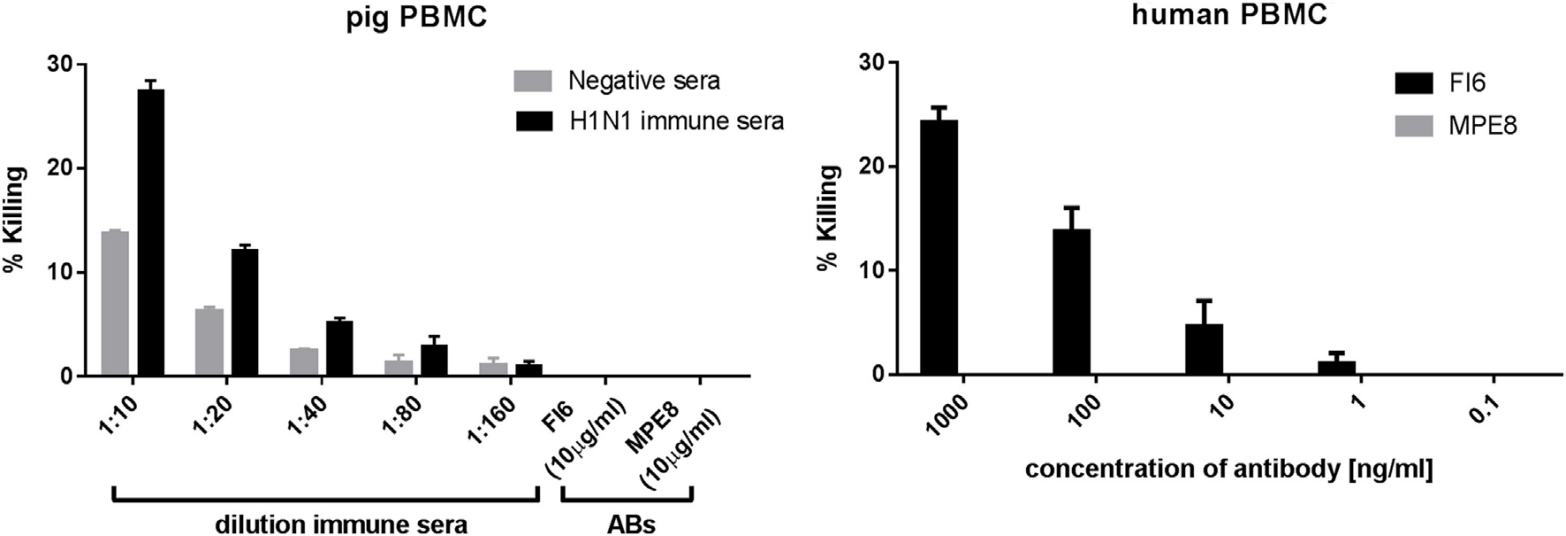
Antibody-dependent cytotoxicity (ADCC) activity of pig and human PBMC. Madin–Darby canine kidney cells expressing H1 HA were incubated with FI6, MPE8, immune, or normal pig sera in the presence of either pig or human PBMC. ADCC was measured in triplicate by lactate dehydrogenase release. FI6 and MPE8 mAbs were used at 10 µg/ml (left panel) or at a concentration range from 0.1 to 1,000 ng/ml (right panel). Representative of three experiments.

Finally, to determine whether FI6 can mediate ADCC in pigs we evaluated killing by LDH release from MDCK cells stably transfected with H1 HA. As previously described, FI6 was able to mediate ADCC with human PBMC as effector cells (Figure 6), but not with pig PBMC. Immune pig serum from influenza infected or immunized pigs gave specific killing.

These results suggest that the failure of FI6 to protect against influenza infection in pigs is most likely due to the inability of FI6 to bind pig FcR and mediate ADCC, and possibly other effector functions (e.g., ADCP).

### Fc Binding Sites in Human, Mouse, and Pig FcγRs and IgG Subclasses

**Figure 7:**
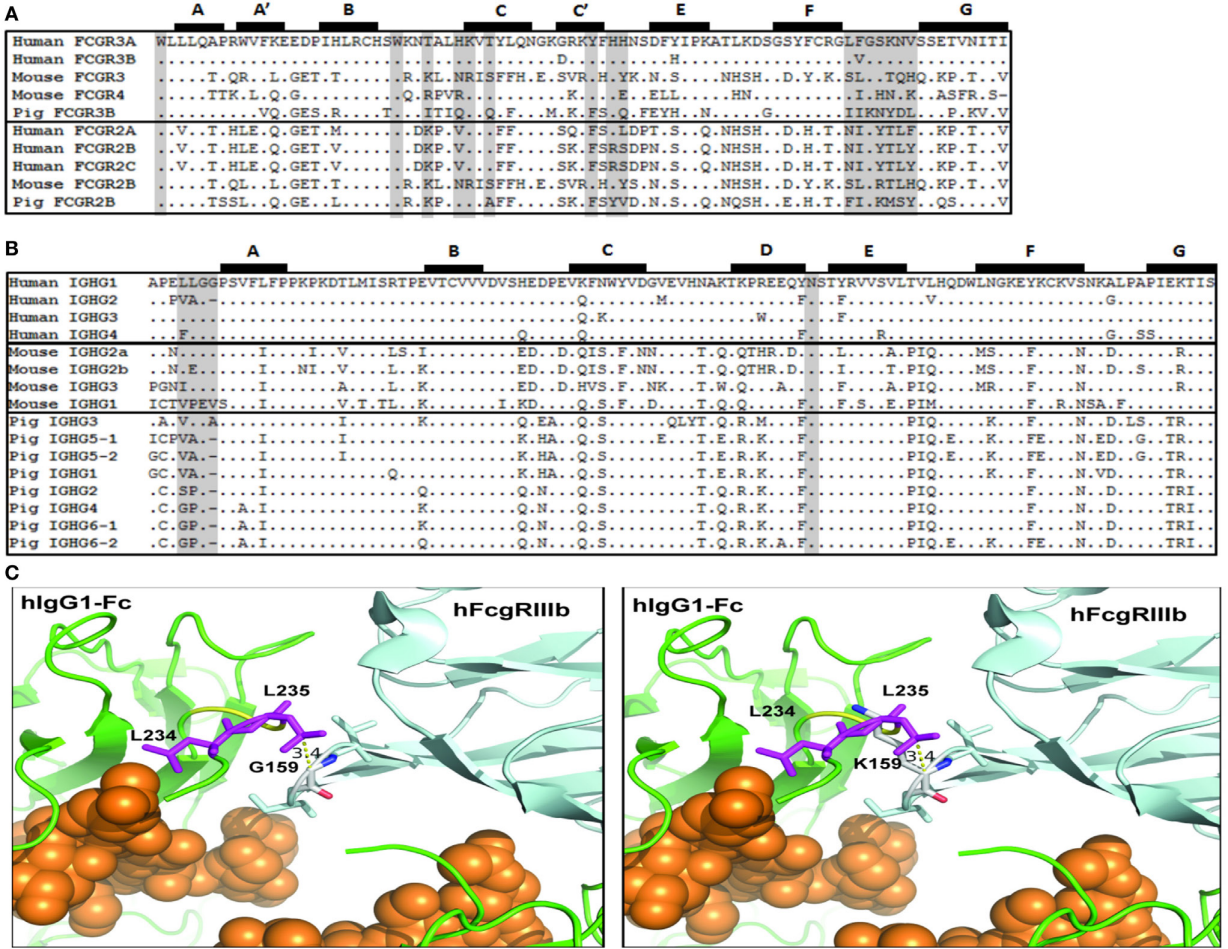
Putative amino acid sequence alignment of FcγR and IgG subclasses. **(A)** Second IgG domain of human, mouse, and pig Fcγ receptors. **(B)** Lower hinge and CH2 domain of human, mouse, and pig IgG subclasses. Previously reported Fc-FcγR contact sites are shaded. Beta-strands are labeled and shown at top. **(C)** Left. Model of the interaction of the Fc region of human IgG1 (green) with human FcgRIIIb (light blue) (pdb, 1t83). Fc-bound glycans are shown as orange spheres. The L234 and L235 Fc residues (purple) and the G159 residue of the FcγRIIIb FG loop are identified. Right. Modeling of the positioning of the porcine K159 residue in the FG loop and its clash with the Fc residue L235.

The apparent failure of FI6 to interact functionally with pig FcR led us to compare the putative binding sites on both the FcγRs and the Fc portion of IgG. Importantly, crystallographic analyses of hIgG complexed with human FcγRIII and the structure for human FcγRI have elucidated the important contact sites for this interaction (33–36). On the receptor, the Fc contact sites are spread across the second immunoglobulin domain, and most notably in the BC, C’E, and FG loops (Figure 7A). Comparison of known mouse, human, and pig FcγR sequences revealed species-specific variation within these regions, and do not immediately suggest that mouse FcγRs would have greater affinity for hIgG than the pig (Figure 7A). However, it has been shown that human IgG1 binds to mouse FcgRIV and effectively induces ADCC and ADCP with mouse NK cells, mouse polymorphonuclear leukocytes, and mouse macrophages (37).

Among the hIgG subclasses, hIgG1 and hIgG3 bind most strongly to FcγRs; whereas, hIgG2 and hIgG4 bind either poorly or not at all (38). Notably on the Fc portion of the antibody, the lower hinge region, the hinge-proximal portion of the CH2 domain, and an N-linked glycosylation site in CH2 are implica-ted in Fc binding. In particular, the motif “LLGG” in the lower hinge is believed to play a crucial role (33, 39, 40). Indeed, mutation of this motif in hIgG1 to either “VLGG” or “LAGG” was previously shown to reduce or prevent binding to FcγRI, respectively (41). In addition, the mutation of residues, L234 and L235 to alanine to generate the so-called LALA mutant abrogates binding to all FcR and C1q (42). Conversely, mutation of hIgG2 from “VAG” to “LLGG” and of hIgG4 from “FLGG” to “LLGG” restored FcγRI binding to levels comparable to hIgG1 (41). Interestingly, mice have fully conserved this motif in mIgG2a, and have similar motifs in mIgG2b and mIgG3 (“LEGG” and “ILGG,” respectively) (Figure 7B). Of the porcine IgGs, however, pIgG3 is most similar (“VLGA”), whereas the rest of the subclasses lack this motif, and are generally more similar to hIgG2 in this region. Thus, the presence of the canonical (“LLGG”) FcγR binding motif in both human and mouse IgG, but not in pig IgG, suggests that porcine FcγRs recognize the Fc portion of IgG differently than in humans and mice. Structural analysis revealed that the LLGG motif of human IgG1 interacts with hydrophobic residues (LVG) in the FG loop of the human FcγRIIIB. Similarly, hydrophobic residues are found in human FcγRIIIB and mouse FcγRIV (LFG and LIG, respectively). Conversely, the pig FcγRIII carries the IIK motif in the FG loop. The analysis of the interaction of the Fc of human IgG1 with human FcγRIIIB indicates that the presence of a lysin at position 159, as found in the porcine FcγRIIIB, would clash with L235, thus interfering with the favorable interaction of the LLGG motif of human IgG1 with the FG loop required for FcγRIII binding (Figure 7C). This observation might explain the lack of binding of human IgG1 FI6 to pig PBMCs. Of note, the hinge regions of all pig IgGs (except for pig IGHG3) are shorter as compared to human IgG1, a finding that might suggest a different modality of interaction of the pig Fcs with the cognate FG loop of porcine FcγRIII.

## Discussion

Our data shows that therapeutic administration of the broadly neutralizing FI6 antibody either I.V. or by aerosol to pigs did not result in exacerbation of disease. Aerosol delivery of FI6 was the only treatment to reduce gross pathology significantly, although viral titers in nasals swabs or BAL were unchanged. We further demonstrated that the pig Fc receptors do not bind human IgG1 and that FI6 did not mediate ADCC with pig PBMC, suggesting that the pig is an inappropriate model to evaluate human IgG1 antibodies.

Previous studies have shown that all neutralizing and non-neutralizing anti-HA (and anti-neuraminidase) mAbs that recognize a breadth of influenza strains require FcγRs for protection *in vivo* (4, 9, 32), while strain-specific mAbs did not. This suggests that the *in vitro* neutralization mechanism of broadly neutralizing mAbs such as inhibition of viral fusion or egress, do not dominate *in vivo* at the doses tested. Our results clearly demonstrate, therefore, that *in vitro* neutralizing antibody responses are not a robust correlate of protection for the control of influenza virus infection and pathology in a natural host model.

There are limited studies describing porcine FcγRs. Although there is obvious overall similarity to their human and mouse counterparts, some FcR in domestic animals are unusual, perhaps most notably bovine Fcγ2R, which although related to other mammalian FcγRs, belongs to a novel gene family and porcine FcγRIIIA, which associates with a molecule that contains significant homology to the cathelin family of antimicrobial proteins (43, 44). Furthermore, the conservation of FcγR binding sites in human and mouse IgG, but not in pig IgG, is consistent with our findings. Clearly differences in interaction with IgG subclasses, cell type, and tissue-specific expression, as well as species differences should be considered when using these models for *in vivo* evaluation of therapeutic mAbs. Substituting the human Fc portion of the FI6 antibody with a pig Fc would provide definitive proof of the importance of Fc binding and ADCC for therapeutic efficacy of FI6.

It is clear that the delivery of FI6 did not cause pathology or exacerbation of disease as described by Khurana et al. (16). In their study, the pigs were immunized with a whole irradiation inactivated, adjuvanted H1N2 (human-like virus), and challenged with a different pdmH1N109 strain. The authors suggested that the vaccine-induced anti-HA stem antibodies facilitated a conformational change in HA that enhanced its fusion and increased virus entry into cells *in vitro*. Nevertheless because FI6 does not engage FcR-mediated effector mechanisms in pigs, it is still possible that these might contribute to VAERD, for example, by massive killing of infected cells, leading to inflammation and pathology.

In summary, our data show that therapeutic administration of FI6 or a control, either I.V. or by aerosol to pigs did not exacerbate disease. Aerosol delivery is an effective means of administration for therapeutic mAbs in large animals and possibly humans. FI6 does not bind to pig Fc receptors or mediate ADCC, confirming previous evidence that ADCC is an important mechanism for protection by anti-stem Ab *in vivo*.

## Data Availability Statement

All datasets for this study are included in the manuscript and the supplementary files.

## Ethics Statement

Animal experiments were approved by the Pirbright Institute ethics committee according to the UK Animal (Scientific Pro-cedures) Act 1986.

## Author Contributions

ET, SM, JH, BH, DC, and BC designed and performed the experiments and analyzed the data. EP collected pathology samples and FS performed the pathological analysis. EC, GA, and BG produced the FI6 antibody and provided advice on ADCC. JS provided sequence alignments. AT and PR provided reagents for and crucial advice on microneutralization assays. SM, ET, and DC wrote and edited the paper.

## Conflict of Interest Statement

DC, EC, GA, and BG are employees of Humabs Biomed, a company that develops anti-infective human monoclonal antibodies. All other authors declare no competing interests.
